# Ecological role of vertebrate scavengers in urban ecosystems in the UK

**DOI:** 10.1002/ece3.2414

**Published:** 2016-09-09

**Authors:** Richard Inger, Daniel T. C. Cox, Esra Per, Briony A. Norton, Kevin J. Gaston

**Affiliations:** ^1^ Environment and Sustainability Institute University of Exeter Penryn UK; ^2^ Faculty of Science Biology Department Gazi University Teknikokullar Ankara Turkey; ^3^ Department of Animal and Plant Sciences University of Sheffield Sheffield UK

**Keywords:** carcass, carcass removal, carrion, carrion crow, ecosystem services, Eurasian magpie, European red fox, scavenger, scavenging, urban

## Abstract

Recent research has demonstrated how scavenging, the act of consuming dead animals, plays a key role in ecosystem structure, functioning, and stability. A growing number of studies suggest that vertebrate scavengers also provide key ecosystem services, the benefits humans gain from the natural world, particularly in the removal of carcasses from the environment. An increasing proportion of the human population is now residing in cities and towns, many of which, despite being highly altered environments, contain significant wildlife populations, and so animal carcasses. Indeed, non‐predation fatalities may be higher within urban than natural environments. Despite this, the fate of carcasses in urban environments and the role vertebrate scavengers play in their removal have not been determined. In this study, we quantify the role of vertebrate scavengers in urban environments in three towns in the UK. Using experimentally deployed rat carcasses and rapid fire motion‐triggered cameras, we determined which species were scavenging and how removal of carcass biomass was partitioned between them. Of the 63 experimental carcasses deployed, vertebrate scavenger activity was detected at 67%. There was a significantly greater depletion in carcass biomass in the presence (mean loss of 194 g) than absence (mean loss of 14 g) of scavengers. Scavenger activity was restricted to three species, Carrion crows *Corvus corone*, Eurasian magpies *Pica pica*, and European red foxes *Vulpes vulpes*. From behavioral analysis, we estimated that a maximum of 73% of the carcass biomass was removed by vertebrate scavengers. Despite having low species richness, the urban scavenger community in our urban study system removed a similar proportion of carcasses to those reported in more pristine environments. Vertebrate scavengers are providing a key urban ecosystem service in terms of carcass removal. This service is, however, often overlooked, and the species that provide it are among some of the most disliked and persecuted.

## 1 Introduction

Energy transfer between trophic levels is a fundamental process in ecology and commonly results from one organism consuming another. This is generally believed to be achieved by predation; however, many predators are also scavengers. While vultures (Accipitridae and Cathartidae) are the only obligate terrestrial vertebrate scavengers, facultative scavenging (not relying on scavenging to survive and reproduce but utilizing carrion when it is available) is phylogenetically and geographically widespread, and may in some cases be favored over predation (Foltan, Sheppard, Konvicka, & Symondson, 2005). Fresh carrion represents a resource equal in value to that acquired by predation, but the risks and energy expenditure associated with scavenging are far lower. Moreover, in at least some systems, predation is not the major form of prey mortality; hence, carrion is likely to be a relatively abundant resource (DeVault, Rhodes, & Shivik, 2003). Indeed, the importance of scavenging has potentially been vastly underestimated in food web research, which may have led both to underestimates of energy flow into higher trophic levels and overestimates of its flow into “brown” decomposition channels (Wilson & Wolkovich, 2011).

Increasing evidence suggests that vertebrate scavengers also provide key ecosystem services, the benefits humans gain from the natural world (Moleón, Sánchez‐Zapata, Selva, Donázar, & Owen‐Smith, 2014; Şekercioğlu, Daily, & Ehrlich, 2004). Humans and vertebrate scavengers have been interdependent for many thousands of years (Moleón, Sánchez‐Zapata, Margalida et al., 2014). Early humans, who potentially gained a significant proportion of their diet via scavenging, formed competitive or facultative relationships with other scavengers (O'Connell, Hawkes, & Blurton Jones, 1988). As human civilization developed however the role of scavengers became more regulatory; by removing animal carcasses (Markandya et al., 2008; Ogada, Keesing, & Virani, 2011), they played an important hygienic role, eliminating sources of toxins and pathogens.

Vertebrate scavengers provide three distinct ecosystem services (Beasley, Olson, & DeVault, 2015). First, they increase connectivity and hence stability within food webs (Rooney, McCann, Gellner, & Moore, 2006; Wilson & Wolkovich, 2011). Second, scavengers can act to distribute nutrients both within and across the borders of ecosystems (Helfield & Naiman, 2001; Hewson, 1995; Huijbers, Schlacher, Schoeman, Weston, & Connolly, 2013; Reimchen, Mathewson, Hocking, Moran, & Harris, 2002; Schlacher et al., 2015). Third, and arguably providing the most direct benefits to humans, are sanitary benefits associated with the removal of carcasses from the environment. Unscavenged carcasses soon become reservoirs for potentially dangerous putrefactive bacteria (Ortiz & Smith, 1994; Vass, 2001) and zoonotic pathogens with potentially fatal impacts for humans (Monroe et al., 2015), livestock (Sterne & Wentzel, 1949), and wildlife (Reed & Rocke, 1992). By removing carcasses from the environment before putrefaction, scavengers prevent the build‐up of toxins in the environment and remove potential disease reservoirs. The importance of scavengers as ecosystem service providers can be demonstrated with examples from across the globe. In India, major anthropogenically induced declines in vulture numbers led to increased numbers of feral dogs and rats, which are reservoirs of rabies and leptospirosis, resulting in an estimated cost to the government of around £10 billion per year in health costs (Markandya et al., 2008). After the outbreak of bovine spongiform encephalopathy in 2001, European Union regulations forced farmers in southern Europe to remove in a more controlled manner carcasses which had previously been removed by scavengers, resulting in increased costs (Margalida & Colomer, 2012), environmental impacts (Morales‐Reyes et al., 2015), and detrimental impacts for vulture populations (Margalida, Donázar, Carrete, & Sánchez‐Zapata, 2010). Scavengers can also be important in removing other organic waste, contributing an additional sanitary regulatory role. For example, Egyptian Vultures (*Neophron percnopterus*) have been estimated to remove over 20% of all organic waste on the island of Socotra, Yemen (Gangoso et al., 2013). The removal of food waste, carcasses, and feces by scavenging vertebrates was once commonplace in many parts of the world, but these tasks have been widely replaced by modern sanitary practices, although they are still undertaken by scavengers in parts of Africa and India (Mundy, Butchart, Ledger, & Piper, 1992; Negro et al., 2002).

Compared to predation, scavenging has been poorly studied, although recent years have seen a surge in interest (Moleón & Sánchez‐Zapata, 2015). The results of this work make it clear that in many habitats the vast majority of carcasses may be removed by vertebrate scavengers (DeVault et al., 2003). The magnitude of vertebrate scavenging is limited by competition with arthropods and decomposers such as microbes and fungi (DeVault, Brisbin, & Rhodes, 2004). Hence, vertebrate scavenging decreases as a proportion of total scavenging/decomposition activity with increasing temperature due to the associated increase in arthropod and decomposer activity. Vertebrate scavenging rates will also be impacted by habitat, as vertebrates are likely to find carcasses more quickly, and hence while they are still fresh enough to consume, in open habitats as opposed to woodland or more complex ones (DeVault, Olson, Beasley, & Rhodes, 2011). The composition of the vertebrate scavenger community will also affect the extent to which scavengers monopolize carrion over invertebrate decomposers. The majority of terrestrial scavenging is carried out by birds and mammals (Mateo‐Tomás et al., 2015), with birds having the advantage of flight to help them locate fresh carcasses more quickly (Ruxton & Houston, 2004). Most birds are however diurnal; therefore, mammals should be expected to consume more carrion during the night.

The key role of scavengers is becoming increasing clear, although most studies have focused on natural habitats (DeVault et al., 2003 and references within; Mateo‐Tomás et al., 2015) or agricultural land (DeVault et al., 2011). The majority of people however now live in urban centers, which, despite being highly altered habitats, may also contain a significant abundance of wildlife (Fuller, Tratalos, & Gaston, 2009). The presence of wildlife means there will also be wildlife carcasses in closer proximity to people than in more natural habitats. While some of these carcasses will be anthropogenically removed, for example, if they present a road safety hazard, the vast majority are removed by scavengers (Slater, 2002; Teixeira, Coelho, Esperandio, & Kindel, 2013), providing they can be accessed. Previous work suggests that scavenger search times and hence carcass removal rates are influenced by the level of anthropogenic habitat alteration. For example, scavenging rates are higher in agricultural habitats when compared to more pristine habitats. Therefore, it might be expected that scavenging rate will be influenced by the amount of high‐quality green space and levels of fragmentation within urban environments. The role of scavengers in urban environments has rarely been considered or quantified, despite the fact they are likely to play a critical role in terms of nutrient recycling, carcass removal, and its associated hygiene benefits.

Here, we explore the role of vertebrate scavengers in urban environments. Specifically, we quantify the proportion of carcasses and carcass biomass removed by vertebrates and how this is partitioned between different species using experimentally deployed carcasses secured in view of motion‐triggered cameras. We test the hypotheses that (1) vertebrate scavengers remove the majority of carcass biomass in urban environments; (2) generally, scavenging rates are higher for birds than for mammals because of their superior search capabilities; (3) scavenging is temporally partitioned, with birds providing scavenging in the daytime and mammals at night; and (4) scavenging rates are impacted by characteristics of the urban environment, being highest in areas with larger amounts of green space and lower levels of fragmentation.

## 2 Materials and Methods

### 2.1 Field methods

Fieldwork was carried out between May and September 2014. Nine experimental sites were located within three adjacent towns in the UK: Milton Keynes (52°02′N, 0°45′W; area = 89 km^2^; population = 230,000; *n* = 5), Bedford (N52°58′N, 0°28′W; area = 24.8 km^2^; population = 106,940; *n* = 3), and Luton (51°53′N, 0°25′W; area = 50.7 km^2^; population = 258,018; *n* = 1). Milton Keynes is the least fragmented town, with high levels of connected green spaces. Luton has an intermediate level of fragmentation and continuous green space, while Bedford is the most fragmented. Sites were all nonwoodland green spaces closed to public access but accessible by potential vertebrate scavengers. Three sites were only accessible by avian scavengers (rooftop locations, one in Milton Keynes, two in Bedford). A minimum of 1 week was allowed before sites were reused between experiments. Each experimental replicate comprised a previously frozen and recently defrosted commercially purchased rat carcass (250–300 g, Live Foods Direct, http://www.livefoodsdirect.co.uk), which was used as carrion. Each rat was weighed to the nearest 1 g, placed onto a wooden platform, and a Reconyx HC 600 Hyperfire camera trap was placed approximately 2 m in front of the carcass such that it was in the movement detection bands. Cameras were set to take color images by day and infrared monochrome images by night. Trigger speed was 0.2 s with a reset speed of approximately 0.5 s, meaning the camera produced a series of images of all animals attending the carcass, provided it detected movement. Rats were secured to the platform with two nails to ensure the carrion remained within the view of the camera trap in order to maximize the recording of scavenging behavior. The experimental setup was left for between 2 and 4 days dependent on logistic constraints, after which data from the camera trap were removed. The remains of the rat were weighed before being disposed off. Experiments were repeated at each site between four and nine times (mean = 7). To estimate biomass loss not due to vertebrate scavenger, we also deployed ten control carcasses. These were placed in metal cages (mesh size approx. 1 × 3 cm^2^) with solid plastic bases that allowed invertebrates but prevented vertebrates from gaining access. Controls were placed in existing experimental sites to ensure matching habitats, and left out for the same time period as the experimental carcasses.

### 2.2 Analysis of scavenger activity

Camera trap photographs were examined individually and sequentially for the presence of any vertebrate animal. The time between an animal initially being recorded by the camera until it could no longer be seen was recorded as a single observation, the unit of replication in the study. Once the animal left the view of the camera, we had no means of knowing whether a subsequent visit by the same species was the same or a different individual; hence, we have no information on the number of individuals of a particular species for any particular experiment.

For each observation, we recorded the animal species (or higher taxonomic level if species identification was not possible), the temperature (recorded by the camera), and one of five behavioral interactions with the carcass: “None”—animal had no interaction with the carcass; “Looking”—animal looking in the general direction of the carcass; “Examining”—animal close to (approx. <2 rat lengths from) the carcass and looking/sniffing/probing at the carcass; “Eating”—animal consuming the carcass; and “Removing”—animal removing the whole carcass from the experiment. Should the interaction change, for example, from “Examining” to “Eating”, then the new interaction was recorded as a new observation.

Each behavioral observation was categorized as occurring during the day or night based on sunrise and sunset figures taken from http://www.sunrise-and-sunset.com. Kernel density functions of activity time for each species were calculated using the “sm” package (Bowman & Azzalini, 2014).

To identify differences between species in time taken to locate carcasses, we measured the time (minutes) taken from the carcass being deployed to the arrival of the first vertebrate scavenger species. These data were used in a generalized mixed‐effects model with a Gamma (link = “inverse”) error structure, with time as the dependent variable, species as a fixed factor, and site identification as a random (intercept) factor.

### 2.3 Overall carcass biomass removed by vertebrate scavengers

To determine the importance of vertebrate scavengers in the removal of carrion, we used a linear mixed‐effects model with carcass weight loss during the course of each experimental replicate as the dependent variable, with a Gaussian error structure (link = “identity”). The presence or absence of vertebrate scavengers at carcasses (defined using camera trap data) was considered as a two‐level factor. Whether nonavian scavengers had access to the site was included as a two‐level factor, monthly mean max temperature (Met Office data, Cambridge NIAB station, temperature from the cameras was not available for all replicates) was included as a covariate, the total time the carcass was deployed was included as a covariate, and deployment time was also included as a covariate. To account for local site effects, site identification and town (site nested within town) were included as random (intercept) factors. To account for temporal autocorrelation, date of deployment of the carcass was incorporated as a random (intercept) factor.

### 2.4 Proportion of carcass biomass consumed by each scavenger species

For each replicate, data from the camera trap analysis were used to calculate the total time that each scavenging species was observed eating the carcass. From this, we calculated the proportion of scavenging behavior (“Eating”) that could be attributed to each species. For each carcass, the total biomass lost during the course of the experiment was first adjusted to take into account loss due to causes other than vertebrate scavenger activity, based on mass loss data from the controls and carcasses where no scavenger behavior was recorded. The remaining mass loss was then attributed to each scavenging species assuming that (1) biomass removed was proportional to time spent feeding; and (2) biomass removed was proportional to body mass of the scavenger. Avian mean body masses were obtained from the British Trust for Ornithology (2015a,b), and mammalian body masses from Sillero‐Zubiri, Hoffman, and MacDonald (2004).

### 2.5 Statistical methods

Residuals from models were visually checked to verify the error structure used. All models were fitted with the R (v3.0.2) language and environment (R core team 2012), using the package “lme4” (Bates, Bolker, Maechler, & Walker, 2013). *F* and *p* values were calculated using Satterthwaite (1946) approximations to determine denominator degrees of freedom in package “lmerTest” (Kuznetsova, Brockhoff, & Christensen, 2013). To evaluate the variance explained, we calculated *R* ^2^ values of the global model, that is, the model containing all the parameters of interest, using the methods of Nakagawa and Schielzeth (2013). We calculated $R_{\mathrm{GLMM}(m)}^{2}$, the marginal R^2^ which describes the variance explained by the fixed factors, and $R_{\mathrm{GLMM}(c)}^{2}$, the conditional *R* ^2^ which is concerned with the variance explained by both the fixed and random factors.

## 3 Results

We deployed 63 experimental rat carcasses between 12 May 2014 and 3 September 2014: 35 in Milton Keynes, 24 in Bedford, and four in Luton. Camera traps took 67487 images from which 2212 observations from 21 vertebrate species (Fig. S1, Table S1) were recorded at 56 different carcasses (89% of the total).

### 3.1 Overall carcass biomass removed by vertebrate scavengers

Scavenging activity was detected at 42 carcasses (67%), and there was a significant difference (*t* = 3.75, *p* < .001) in carcass mass loss between carcasses attended by vertebrate scavengers (mean = 194.72 g, SD = 111.86 g) and those with no vertebrate scavengers (mean = 13.71 g, SD = 17.28 g). When considered as a percentage of the original mass (total consumption = 100%), mean mass loss in the presence of vertebrate scavengers was 66% (SD = 37%), compared to 5% (SD = 5%) in the absence of vertebrate scavengers. There were no significant differences in mass loss with temperature (*t *= −0.625, *p* = .537), whether only birds, or both birds and mammals could access the carcass (*t* = 1.90, *p* = .07), time of deployment (*t* = −0.805, *p* = .426), or in relation to the total deployment time of the carcass (*t* = −0.675, *p* = .537). The general linear model accounted for 57% of the variation in mass loss, with 22% being explained by the fixed effects (See Table 1 for full model details).

**Table 1:** Parameter estimates for factors effecting the loss of carrion biomass. “No” is base level for Scavenger activity. “No” is base level for Access to foxes. “***” = significant at 0.001

	Estimate	SE	df	*t* value	*p*‐value
Intercept	215.795	184.448	31.560	1.170	.250
Scavenger Activity	109.998	29.286	46.160	3.756	<0.001***
Rat deployment time	−8.785	13.023	36.260	−0.675	.504
Access to Foxes	83.635	44.076	23.640	1.898	.070
Temperature	−4.331	6.924	26.090	−0.625	.537
Deployment Time	−4.285	5.321	37.090	−0.805	.426

Only carrion crows (*Corvus corone,* hereafter crows), Eurasian magpies (*Pica pica,* hereafter magpies), and European red foxes (*Vulpes vulpes,* hereafter foxes) were recorded scavenging the carcasses, either by eating them in situ, or by removing whole carcasses from the experimental sites. Despite the carcasses being secured to the observation platforms, 17 were eventually removed by persistent animals, in particular foxes. Carcasses which were removed from the view of the camera were assumed then to be fully consumed elsewhere (Moleón, Sánchez‐Zapata, Sebastián‐González, and Owen‐Smith, 2015). In total, 1,672 observations were made of one of these three scavenging species at the carcasses. In 742 cases (44% of the total), scavenging behavior was recorded.

**Figure 1:**
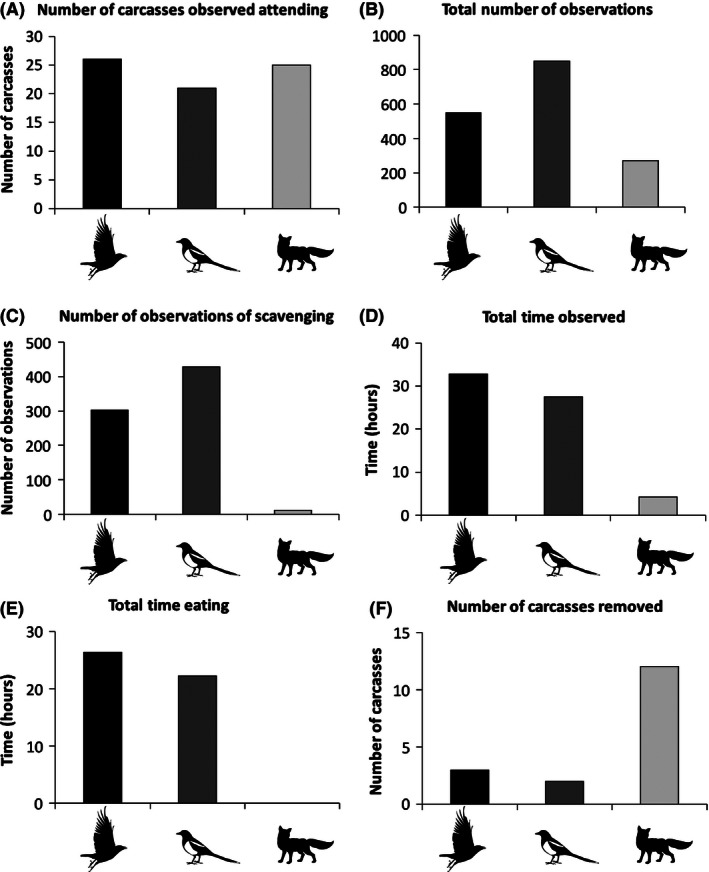
(A) Number of carcasses at which each of the three scavenger species was observed attending. (B) Total number of observations of each species. (C) Total number of observations of scavenging behavior (eating or removing the carcass) for each species. (D) Total time (hours) each species was observed during the study. (E) Total time eating the carcass by each species during the study. (F) Total number of carcasses removed by each species

Crows were the most commonly observed species around carcasses (41% of carcasses), followed by foxes, which were observed at 39% of carcasses, with magpies being seen around 33% of all carcasses (Fig. 1A). Foxes, however, did not have access to three sites, and considering only the sites accessible to them, were observed at 59% of sites. Magpies, despite being found around the smallest proportion of the carcasses, accounted for the greatest number of all observations (51% when only the three scavenging species are considered), followed by crows (33%) and foxes (16%; Fig. 1B). When only scavenging behaviors (Eating and Removing) are considered, magpies accounted for the majority of observations (57%), followed by crows (41%), and only 2% of all scavenging behavior was performed by foxes (Fig. 1C). The total time observed (Fig. 1D) and total time observed Eating (Fig. 1E) were both highest in crows, followed by magpies and foxes. Foxes were never recorded eating the carcasses in situ, but fully removed 12 carcasses, while crows and magpies removed three and two carcasses, respectively (Fig. 1F).

There was very strong time partitioning between avian scavengers, which were observed almost exclusively in the daytime, and foxes, which were observed primarily at night or at dawn or dusk (Fig. S2). Of all observations for crows and magpies, 98% and 99.6%, respectively, were made during the day, while only 9.2% of all fox observations were made during the day.

Foxes were significantly slower in locating carcasses than either crows (*t* = −2.842, *p* = .004) or magpies (*t* = −2.31, *p* = .02). The shortest mean time was for magpies (504 min, SD = 436), followed by crows (683 min, SD = 460) and foxes (755 min, SD = 278).

### 3.2 Proportion of carcass biomass consumed by each scavenger species

**Figure 2:**
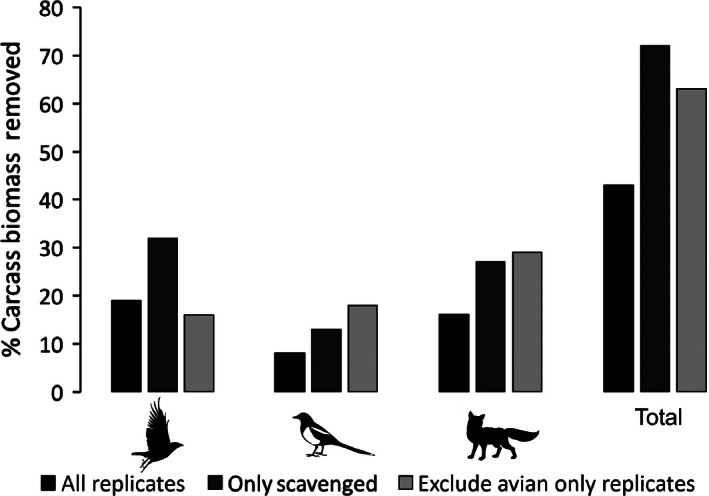
Percentage of carcass biomass consumed by each of the three scavenger species and the total for all species. Different shaded bars represent all experimental replicates, only replicates where scavenger activity was present, and lastly where avian only replicates were also excluded

In total, 18,610 g of carcass biomass was deployed during the course of the experiment. Of this, we estimate that 9.6% was lost to causes other than vertebrate scavenging (as estimated using the control carcasses). Of the remaining 16,823 g, we estimate, based on data from the behavioral analysis, that 8,021 g (43%) can be attributed to vertebrate scavengers, with 3,586 g (19%) being removed by crows, 1,416 g (8%) by magpies, and 3,018 g (16%) by foxes (Fig. 2), while the remaining biomass was unconsumed. When we consider only the carcasses that were scavenged, the biomass deployed was 11,277 g. Of this, we estimate that 32% was consumed by crows, 13% by magpies, and 27% by foxes; a total of 72% (Fig. 2). Foxes did not have access to three sites, and if these are also excluded from the analysis, 16% of the biomass was eaten by crows, 18% by magpies, and 29% by foxes, totaling 63% (Fig. 2). Hence, the total estimated removal of carcass biomass by vertebrates as determined from behavioral analysis from camera images was between 63 and 72%, dependent on which species have access to the carcass. This encompasses the overall biomass loss in the presence of scavengers as determined by weighing the carcass before and after the experiment (66%). This gives confidence that the estimates of carcass removal by each species are robust.

## 4 Discussion

Recent research has highlighted the fundamental role scavenging of carrion can play in ecosystems (Barton, Cunningham, Lindenmayer, & Manning, 2013; Beasley, Olson, & DeVault, 2012; DeVault et al., 2003; Mateo‐Tomás et al., 2015; Moleón & Sánchez‐Zapata, 2015; Moleón, Sánchez‐Zapata, Selva et al., 2014; Pereira, Owen‐Smith, & Moleón, 2014; Wilson & Wolkovich, 2011). However, despite an increasing proportion of the human population residing in cities and towns, and a global increase in urbanized land cover (Seto, Güneralp, & Hutyra, 2012), the role of scavengers in urban ecosystems has received little attention (but see Gangoso et al., 2013). Urban ecosystems may in some cases be richer in ecosystem service provision (Edmondson, Davies, McCormack, Gaston, & Leake, 2014) and biodiversity (Fuller et al., 2009; McKinney, 2008) than surrounding habitats, and are regulated by many of the same ecosystem processes as more pristine habitats, including the removal and recycling of carcasses. In addition, nonpredatory fatalities in urban ecosystems are likely to be of similar or greater importance to those in more pristine ecosystems. For example, window strikes and power line collisions are a major cause of mortality in birds (Hager, Cosentino, & McKay, 2012), and animal–vehicle collisions are a major source of terrestrial vertebrate mortality (Forman & Alexander, 1998), much of which will occur within urban areas. Hence, urban ecosystems are likely to create significant quantities of carcasses, although these quantities and their fates are unknown. Our results represent the first quantification of the role of vertebrate scavengers in carcass consumption within an urban ecosystem.

The majority (67%) of all carcasses in our experiment were, to some extent, scavenged by vertebrates. This is similar to the scavenging rates calculated for natural habitats in North, Central and South America, and Europe (mean = 74%, DeVault et al., 2003). This suggests that vertebrate scavengers play an analogous role in urban ecosystems to that in more natural habitats. A clear caveat here is that we do not know how comparable different ecosystems are in terms of carcass density and carcass size spectra. Indeed, truly to understand the role and importance of scavengers, there is an urgent need to quantify carcass densities and size spectra in a range of habitats and scavenger communities.

Many previous studies have tended to use the percentage of experimental carcasses removed, or to some extent scavenged, as a measure of scavenger efficiency. Recently, more sophisticated studies have reported the impacts on scavenger efficiency of species richness and the presence of obligate scavengers (Sebastián‐González et al., 2013), of carcass size (Moleón et al. 2015) and of scavenger community structure (Sebastián‐González et al., 2016; Selva & Fortuna, 2007). In this study, we calculate estimates of total carcass biomass loss, and how this can be partitioned within the scavenger community. This is important if we are to determine the relative contributions of different members of the scavenger community, as species richness alone may overestimate or underestimate the proportional contribution of each species to total scavenger efficiency, as it is the most common species which are likely to contribute the most overall (Inger, Per, Cox, & Gaston, 2016). Of the total carcass biomass deposited during the experiment, we estimate that 43% was consumed by vertebrates, contrary to our predictions that vertebrates would remove the majority. Of this, we estimate that 27% was consumed by birds (crows—19% & magpies—8%) and 16% by foxes. When, however, we consider only the carcasses that were located by vertebrate scavengers this figure rises to 72% of which 45% was removed by birds (crows—32%, magpies—13%) and 27% by foxes. We suspect that in many habitats the actual carcass removal levels will be closer to the higher estimates, because vertebrate scavengers will have longer to locate the carcasses. Locating a carcass may take a week or more, and they were only left exposed in this experiment for 2–4 days; hence, our estimates are very likely to be less than the true scavenging rates. In the only other comparable study (i.e., conducted in the UK), Putman (1983) found that in the summer months after seven to 8 days vertebrate scavengers had removed 64–90% of all carcasses, and any remains had completely decomposed.

In keeping with our predictions, we found that birds removed the majority of the carcass biomass. Indeed, scavenging birds accounted for the majority of all observations, observations of scavenging, total time observed, and total time scavenging (Fig. 1). Birds have a number of adaptations making them efficient at locating carcasses. By utilizing flight and a keen sense of vision, they can visually search a larger area more quickly and efficiently than mammals (Ruxton & Houston, 2004). Also, they can utilize social information from conspecifics in order to locate carcasses. Social facilitation is well studied in obligate scavenging vultures (Jackson, Ruxton, & Houston, 2008), and information transfer between colonial conspecifics in order to find the best foraging areas has been identified in a number of colonial (Wakefield et al., 2013) and communal roosting species. Raven roosts, for example, can act as centers for the dissemination of information on sources of food including ephemeral sources such as carcasses (Marzluff, Heinrich, & Marzluff, 1996). Finally, it is becoming clear that some birds have a well‐developed sense of smell and can use it to detect food sources (Amo, Jansen, van Dam, Dicke, & Visser, 2013), including carcasses. Both neotropical vultures and nearctic ravens have been shown experimentally to be able to locate carcasses using purely olfactory means (Harriman & Berger, 1986; Houston, 1986).

Despite the advantages birds have, foxes still managed to locate, remove, and (presumably) consume a large proportion of the carcass biomass. Foxes are almost certainly able to detect carcasses by olfactory means, as all encounters with foxes occurred during the night. Indeed, as predicted, there was almost perfect time partitioning between foxes and birds, with foxes scavenging at night and birds foraging during the day. One weakness of our experimental design however, which may have biased our results in favor of avian scavengers, was that carcasses were always deployed in the daytime. Hence, diurnal avian scavengers had an increased probability of locating the carcasses before nocturnal scavengers.

We found that birds were significantly quicker in locating carcasses than foxes, although there are a number of caveats associated with this finding. First, most of the variation explained by the model was due to the location of the carcass, suggesting that carcasses are more easily located in certain habitats. Second, we deployed all the carcasses in this study during the day, which potentially gave the birds a “head start” in locating and consuming them. Levels of habitat fragmentation are known to have an impact on scavenger efficiency with scavengers locating carcasses more easily in fragmented versus more pristine habitats (DeVault et al., 2011). Our study was conducted within three towns with different levels of continuous green space. Milton Keynes has large, well‐connected green spaces, Luton has a more fragmented network of green spaces, and Bedford has the lowest levels of connected green space. Contrary to our predictions, we found no difference in carcass biomass removal or scavenger behavior between the different urban forms. This may be because the three scavenger species are all highly adapted to urban environments (Jerzak, 2001; Scott et al., 2014; Vuorisalo et al., 2003) and can move equally well in green spaces and the built environment.

Carrion is a valuable resource producing intense competition between vertebrate and invertebrate scavengers and microorganisms, and this competition can be strongly influenced by abiotic factors, including temperature (DeVault et al., 2004). Here, we found no effect of temperature on mass loss of the carcasses, or on the behavior of scavengers, likely due to the fact that the mean temperature only varied over a narrow range compared to previous studies (Fig. S4). We did, however, identify a significant change in scavenger behavior with increases in activity around the carcass as the weeks of the experiment progressed, particularly for crows. This may be due, to some extent, to the crows becoming habituated to carcasses being located in a familiar spot. Alternatively, it maybe a function of a decrease in microbial activity as the summer moved into autumn, shifting the competition between microbial and vertebrate scavengers in favor of the vertebrates.

We found a surprisingly low species richness within the scavenger community with only three species displaying scavenging behavior. This was despite there being a number of other species recorded near the carcasses which have previously been recorded as facultative scavengers, including domestic cats and dogs (Jennelle, Samuel, Nolden, & Berkley, 2009), European hedgehog, *Erinaceus europaeus* (Ragg, Mackintosh, & Moller, 2001) and European badger, *Meles meles* (Asprea & De Marinis, 2005), and also brown rats, *Rattus norvegicus,* and sparrowhawks, *Accipiter nisus*. Three species are considerably lower than the scavenger community species richness found in other parts of the world. Mateo‐Tomás et al. (2015) examined nine individual studies from different ecosystems across the globe, from Arctic tundra to temperate forests, Africa savannah, and arid Australian desert, finding between six and 29 scavenging species (mean = 14, SD = 8). All of these studies were however carried out in “natural” ecosystems. These studies also tended to use larger carcasses than those in the current study. Although, given that all the large UK scavengers were already represented in our study, we doubt that the use of larger carcasses would affect our scavenger species richness estimates. Indeed, one previous study using larger carcasses (deer, *Cervus Nippon* and *Capreolus capreolus*, used as proxies for humans in a criminal forensics study), found a scavenger species richness of three, with two species being responsible for 98% of all scavenging events.

The proportion of all scavengers which were birds and mammals in our study was in line with global averages (Mateo‐Tomás et al., 2015), the majority of scavenging species (66.67%, *n* = 2) being birds, compared to a global average of 59.69%. The percentage of mammals recorded in scavenging experiments (33.33%, *n* = 1) also corresponded with global figures with a mean of 37.98% (SD = 12.99).

In conclusion, our results suggest that a relatively depauperate urban scavenger community is able to maintain the same level of ecosystem function as found in much richer non‐urban ecosystems. This service is however not currently valued and is frequently overlooked by ecologists and land managers (Gangoso et al., 2013; Wenny et al., 2011). The wider public also tend not to have favorable views of scavenging species; indeed, the two avian species recorded scavenging in this study are among the most disliked bird species in the UK (Cox & Gaston, 2015), and all three scavenging species can be legally killed as pests. Conversely across the globe, it is becoming increasingly clear that many highly charismatic species of mammals and birds are also scavengers (Moleón & Sánchez‐Zapata, 2015), and are often the focus of targeted conservation schemes. With an increased understanding of the importance of vertebrate scavengers within ecosystems and their role in promoting human well‐being, perhaps we should foster a greater appreciation for these species and the services they provide.

## Funding Information

Scientific and Technological Research Council of Turkey (Grant/Award Number: “Post‐doctoral fellowship”) Natural Environment Research Council (Grant/Award Number: “NE/J015237/1”).

## Conflict of Interest

None declared.

## Data Accessibility

Data will be archived in the NERC Environmental Data Information Centre from mid‐2017.
