# Supplementary material for: Ecological role of vertebrate scavengers in urban ecosystems in the UK
Source: Ecol Evol. 2016 Sep 9;6(19):7015–23. doi: 10.1002/ece3.2414 (PMC5513233; doi:10.1002/ece3.2414)
Supplement: Supplementary file 1 [file ECE3-6-7015-s001.docx]

**SUPPLEMENTARY MATERIALS**

**­Role of Vertebrate Scavengers in Urban Ecosystems**

Richard Inger, Daniel T.C. Cox, Esra Per, Briony A. Norton & Kevin J. Gaston


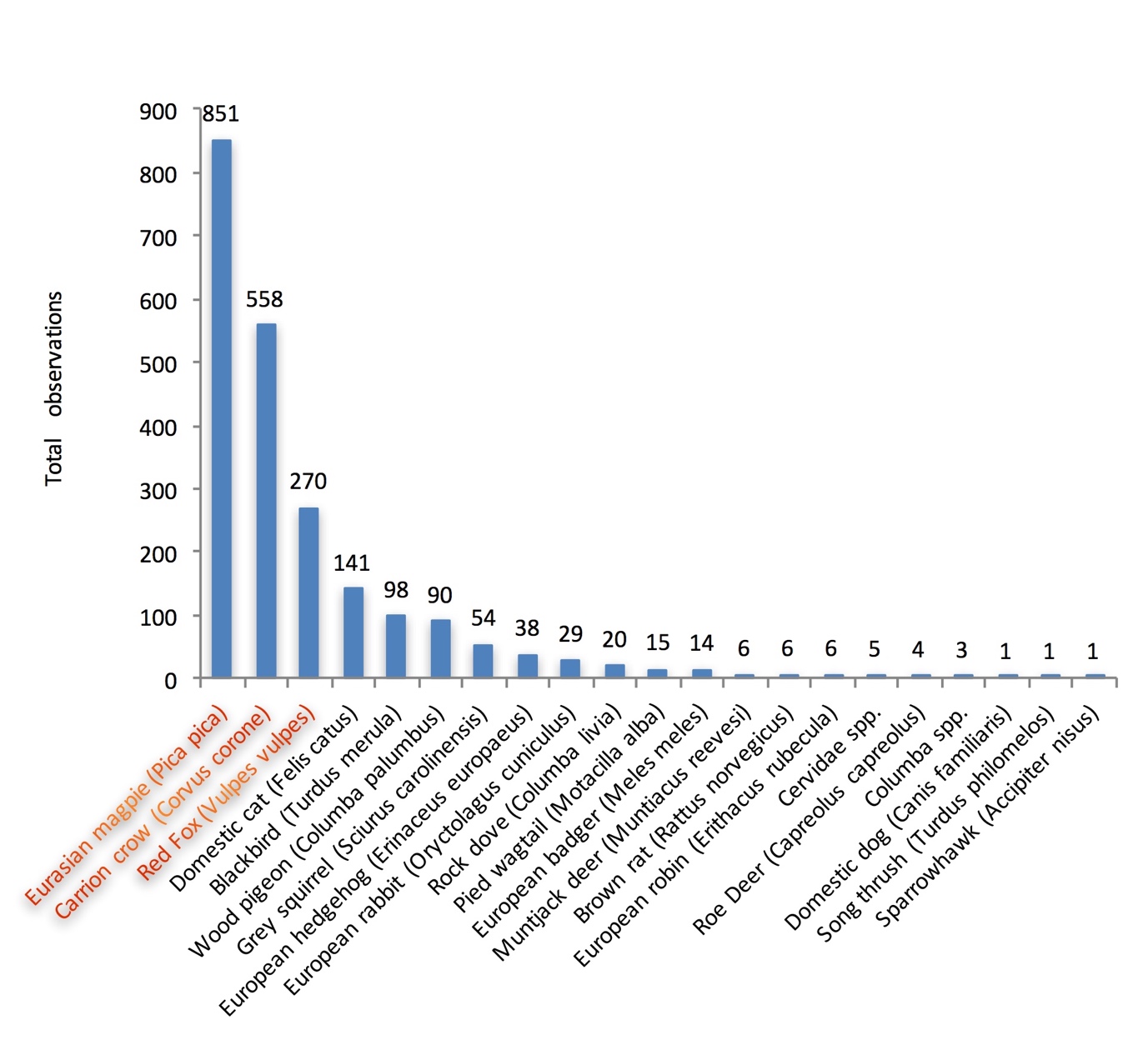


**Figure S1**. Total number of observations of all vertebrates. Scavengers are in red text.

| Species | Bedford | Luton | Milton Keynes | Total |
| --- | --- | --- | --- | --- |
|  |  |  |  |  |
| Magpie | 60 | 35 | 756 | 851 |
| Crow | 345 | 0 | 213 | 558 |
| Fox | 227 | 26 | 17 | 270 |
| Cat | 89 | 0 | 52 | 141 |
| Blackbird | 48 | 15 | 35 | 98 |
| Wood pigeon | 61 | 0 | 29 | 90 |
| Squirrel | 27 | 16 | 11 | 54 |
| Hedgehog | 25 | 0 | 13 | 38 |
| Rabbit | 0 | 0 | 29 | 29 |
| Pigeon | 13 | 0 | 7 | 20 |
| Pied wagtail | 0 | 0 | 15 | 15 |
| Badger | 0 | 14 | 0 | 14 |
| Muntjack deer | 5 | 1 | 0 | 6 |
| Rat | 0 | 0 | 6 | 6 |
| Robin | 0 | 6 | 0 | 6 |
| Deer | 0 | 0 | 5 | 5 |
| Roe Deer | 0 | 0 | 4 | 4 |
| Dove | 0 | 0 | 3 | 3 |
| Dog | 0 | 0 | 1 | 1 |
| Song Thrush | 0 | 1 | 0 | 1 |
| Sparrowhawk | 1 | 0 | 0 | 1 |
| Total | 901 | 114 | 1196 | 2211 |

**Table S1.** Number of observations by species and by town.

**Figure S2.** Kernel density function of timing of observations for crows, magpies and foxes. Shaded areas represent the time from the earliest to latest sunrise and sunset times.
